# Evaluation of the Microbial Profile on the Polydioxanone Membrane and the Collagen Membrane Exposed to Multi-Species Subgingival Biofilm: An In Vitro Study

**DOI:** 10.3390/membranes13120907

**Published:** 2023-12-14

**Authors:** Marcus Vinícius Cintra Moreira, Luciene C. Figueiredo, Marcelo Augusto Ruiz da Cunha Melo, Fabio Hideaki Uyeda, Lucas Daylor Aguiar da Silva, Tatiane Tiemi Macedo, Roberto Sacco, Carlos Fernando Mourão, Jamil A. Shibli, Bruno Bueno-Silva

**Affiliations:** 1 Department of Periodontology, Dental Research Division, Guarulhos University, Guarulhos 07023-070, SP, Brazil; marcuscintra@gmail.com (M.V.C.M.); lucienedefigueiredo@gmail.com (L.C.F.); uyeda_odontologia@hotmail.com (F.H.U.);; 2 Department of Oral Surgery, Faculty of Dentistry, Oral & Craniofacial Sciences, King’s College London, London SE1 9SP, UK; 3 Department of Periodontology, Tufts University School of Dental Medicine, Boston, MA 02111, USA; 4 Departament of Bioscienses, Piracicaba Dental School, University of Campinas, Piracicaba 13414-903, SP, Brazil

**Keywords:** microbial profile, polydioxanone membrane, collagen membrane, subgingival biofilm

## Abstract

Dehiscence in surgeries involving membranes often leads to bacterial contamination, hindering the healing process. This study assessed bacterial colonization on various membrane materials. Polydioxanone (PDO) membranes, with thicknesses of 0.5 mm and 1 mm, and a collagen membrane were examined. Packages containing polystyrene pins were crafted using these membranes, attached to 24-well plates, and exposed to oral bacteria from supra and subgingival biofilm. After a week’s anaerobic incubation, biofilm formation was evaluated using the DNA–DNA hybridization test. Statistical analysis employed the Kruskal–Wallis test with Dunn’s post hoc test. The biofilm on the polystyrene pins covered by the 0.5 mm PDO membrane showed a higher count of certain pathogens. The collagen membrane had a greater total biofilm count on its inner surface compared to both PDO membranes. The external collagen membrane face had a higher total biofilm count than the 0.5 mm PDO membrane. Furthermore, the 1 mm PDO membrane exhibited a greater count of specific pathogens than its 0.5 mm counterpart. In conclusion, the collagen membrane presented more biofilm and pathogens both internally and on its inner surface.

## 1. Introduction

Oral health, a vital sphere of overall health, reflects and impacts general well-being. Among oral afflictions, periodontitis stands out for its high prevalence and associated complications [1]. Originating from a dysbiotic dental biofilm, this pathology is the outcome of the intricate interaction between the host and a vast bacterial community that predominantly establishes itself in subgingival regions, leading to the progressive destruction of dental support tissues [2,3].

The quest for effective therapeutic solutions has led us to explore various regenerative approaches. One of these strategies involves the use of membranes that serve as barriers, promoting and guiding tissue regeneration [4,5]. However, the success of these membranes is not only in their regenerative capacity but also in how they interact with the diversified oral microbiome [6,7,8,9].

Unfortunately, the success of regenerative procedures can be hindered by the presence of specific pathogens, such as *Porphyromonas gingivalis* and *Tannerella forsythensis* [10,11]. It has been observed that bacterial adhesion and the subsequent colonization of exposed membranes can lead to infections in the underlying tissues, resulting in implant failures and ultimately compromising the entire regenerative procedure [11]. Additional studies have shown that specific pathogens, particularly Gram-negative anaerobic bacilli, have a marked predilection for certain membranes, especially those based on e-PTFE [12,13,14].

It is not enough for pathogens to merely adhere to the membranes. Once adhered, pathogens like *Treponema denticola* and *P. gingivalis* have been shown to degrade the membranes, especially those made of collagen, rapidly [15,16,17]. This affinity and subsequent degradation pose a substantial risk for regenerative procedures. Beyond compromising the membranes’ integrity, these bacteria can trigger inflammatory reactions that are counterproductive to tissue regeneration [18,19].

In this context, the need for a meticulous examination of the interaction between regenerative membranes, such as PDO and collagen, and the subgingival biofilm becomes crystal clear. Understanding this dynamic can enable more effective clinical practice and pave the way for the development of new membranes or therapies that maximize periodontal regeneration while simultaneously minimizing the risks associated with bacterial colonization. Thus, this study aims to assess the biofilm profile present on the interior, the external face (oriented towards the periosteum), and the internal part (oriented towards the graft) of the membranes made of the polydioxanone polymer and collagen.

## 2. Materials and Methods

In biofilm formation, the following species were used: *Actinomyces gerencseriae* ATCC 23840, *Actinomyces israelii* ATCC 12102, Actinomyces naeslundii ATCC 12104, *Actinomyces oris* ATCC 43146, *Actinomyces odontolyticus* ATCC 17929, *Veillonella parvula* ATCC 10790, *Streptococcus gordonii* ATCC 10558, *Streptococcus intermedius* ATCC 27335, *Streptococcus* mitis ATCC 49456, *Streptococcus oralis* ATCC 35037, *Streptococcus sanguinis* ATCC 10556, *Streptococcus anginosus* ATCC 33397, *Streptococcus mutans* ATCC 25175, *Aggregatibacter actinomycetemcomitans* ATCC 29523, Capnocytophaga gingivalis ATCC 33624, *Capnocytophaga ochracea* ATCC 33596, *Capnocytophaga sputigena* ATCC 33612, *Eikenella corrodens* ATCC 23834, *Campylobacter gracilis* ATCC 33236, *Campylobacter rectus* ATCC 33238, *Campylobacter showae* ATCC 51146, *Eubacterium nodatum* ATCC 33099, *Eubacterium saburreum* ATCC 33271, *Fusobacterium nucleatum* subsp. *polymorphum* ATCC 10953, *Fusobacterium nucleatum* subsp. *vincentii* ATCC 49256, *Fusobacterium periodonticum* ATCC 33693, *Parvimonas micra* ATCC 33270, *Prevotella intermedia* ATCC 25611, *Streptococcus constellatus* ATCC 27823, *Tannerella forsythia* ATCC 43037, *Porphyromonas gingivalis* ATCC 33277, *Gemella morbillorum* ATCC 27824, *Propionibacterium acnes* ATCC 11827, and *Selenomonas noxia* ATCC 43541.

Most of the species, including *Actinomyces* subsp., *Streptococcus* subsp., and Fusobacterium subsp., were cultivated on tryptic soy agar supplemented with 5% of sheep’s blood under anaerobic conditions (85% nitrogen, 10% carbon dioxide, and 5% hydrogen). *P. gingivalis* was grown on tryptic soy agar with a yeast extract enriched with 1% hemin, 5% menadione, and 5% sheep’s blood, while T. forsythia was cultivated on tryptic soy agar with a yeast extract enriched with 1% hemin, 5% menadione, 5% sheep’s blood, and 1% N-acetylmuramic acid. After 48 h of growth, all species were transferred to falcon tubes with a BHI culture medium (Becton Dickinson, Sparks, MD, USA) supplemented with 1% hemin.

Following overnight growth in BHI broth with 1% hemin, the optical density (OD) at 600 nm was adjusted to 0.1, corresponding to about 108 cells/mL for each of the species. Individual cell suspensions for each species were diluted to 107 cells/mL and adjusted for their respective cell sizes. Aliquots of 100 µL containing 106 cells for each of the species were mixed to obtain a final biofilm inoculum. Twelve milliliters and seven hundred microliters of BHI broth with 1% hemin and 5% sheep’s blood were added to obtain a final volume of 15 mL of inoculum containing ~1 × 104 cells of each of the species, except *P. gingivalis* and *P. intermedia*, which were added at quantities of 2 × 104 cells.

From this inoculum, 2 mL was placed into the wells of 24-well plates, with each well containing a membrane with an acrylic pin inside. After 72 h of incubation, the membranes were transferred to new 24-well plates with a fresh broth (BHI broth with 1% hemin and 5% sheep’s blood) and were kept in this plate for an additional 4 days. On the seventh day, the biofilms were collected, as described below.

### 2.1. Membranes Preparation

#### 2.1.1. Plenum Membranes

The experiment used two types of PDO membranes (Plenum Bioengenharia, Jundiai, SP, Brazil), PDO 0.5 and PDO 1, which were 0.5 mm and 1 mm thick, respectively. Three membranes of each type were folded equally, and a rough polystyrene pin was placed inside. The membranes were sealed only along the edges with a suture to ensure that their permeability was not affected during the experiment.

#### 2.1.2. Bio-Guide Membrane

Two bio-guide membranes (Geistlich Pharma AG, Bahnhofstrasse, Wolhusen, Switzerland) were used in the study. They were prepared in the same way as the membranes mentioned earlier, with the following single important difference: this membrane has different surface characteristics on both sides. As a result, the more porous side was placed facing the graft to allow for better contact with the polystyrene pin, while the smoother side, which the manufacturer recommended to be in contact with the periosteum, was placed directly in contact with the biofilm.

### 2.2. Membrane Stamps

To prevent the loss of data regarding what might have colonized internally within each membrane, the internal part of each was put into contact with an agar culture medium to cultivate each bacterial biotype present and identify which species colonized or surpassed each type of membrane. The external part, in contact with the culture medium, was put in contact with the agar on the upper part of the Petri dish and was also used as a stamp.

Notably, to make the stamps, the PDO 0.5 AND 1 membranes, which were more intact, made the technique easier. The collagen membrane, after a week in contact with the multi-species biofilm, exhibited more decomposition, less integrity (perforations), and appeared as a single mass with the pin inside.

### 2.3. DNA–DNA Hybridization (Checkboard DNA–DNA)

Following the growth of bacteria on the agar according to the transfer procedure (“stamp”) of the internal and external faces of the used membranes, the bacteria were collected with a platinum loop and transferred to Eppendorf tubes containing 100 µL of the TE buffer (Tris 10 mM-HCl, EDTA 1 mM, pH 7.6), and then 100 µL of 0.5 M NaOH was added.

Bacteria that formed a biofilm on the polystyrene pins added inside the membranes were also collected. After the biofilm formation period (7 days), these pins were transferred to Eppendorf tubes containing 100 µL of the TE buffer, sonicated for 10 min to remove the biofilm from the pin’s surface, and then 100 µL of 0.5 M NaOH was added.

Tubes containing the final solution were boiled for 10 min, and the solution was neutralized by adding 0.8 mL of 5 M of ammonium acetate. The samples were then individually analyzed regarding the presence and quantity of the 33 bacterial species using the DNA–DNA hybridization technique.

Each suspension containing the free DNA of each sample’s biofilm was deposited in one of the channels of the Minislot (Immunetics, Cambridge, MA, USA), thereby concentrating on a nylon membrane. The last two channels of the Minislot were occupied by controls containing a mix of the DNA of the investigated microorganism species at concentrations corresponding to 10^5^ and 10^6^ cells [17,18,19]. The membrane was then removed from the Minislot, and the concentrated DNA was fixed via heating in an oven at 120 °C for 20 min. The membrane was then placed in the Miniblotter (Immunetics) with the DNA lines perpendicular to the apparatus’s channels. In each Miniblotter channel, a single bacterial species DNA probe was added. Hybridization occurred for a minimum of 20 h at 42 °C.

After hybridization, the membranes were washed to remove the probes that did not completely hybridize. The membrane was then immersed for 1 h in a solution containing 1% maleic acid (C4H4O4), 3 M NaCl, 0.2 M NaOH, 0.3% Tween 20, 0.5% casein, pH 8.0, and immediately after, for 30 min in the same solution, containing the anti-digoxigenin antibody conjugated to alkaline phosphatase. Finally, the DNA probes were detected using a specific antibody for digoxigenin conjugated with alkaline phosphatase. The signals were detected using the AttoPhos substrate (Amersham Life Sciences, Arlington Heights, IL, USA), and the results were read using Typhoon Trio Plus (Molecular Dynamics, Sunnyvale, CA, USA). Signals obtained with the Typhoon Trio were converted into absolute counts by comparison with the standards on the same membrane.

### 2.4. Statistical Analysis

Statistical analysis was performed using the Kruskal–Wallis test and Dunn’s post hoc test (*p* ≤ 0.05).

## 3. Results

This study found that the collagen and PDO 0.5 membranes had *S. gordonii*, *F. periodonticum*, *T. forsythia*, and *S. anginosus bacteria.* The only bacteria with a statistically significant difference (*p* ≤ 0.05) between the pin inside the collagen membrane and the PDO 1 membrane was *S. sanguinis*, which had a higher count on the pin inside the collagen membrane. Furthermore, *P. intermedia* was the only bacteria that showed a statistically significant difference (*p* ≤ 0.05) in the counts between the pin inside the collagen membrane and the pin inside both the PDO 0.5 and PDO 1 membranes, with higher counts on the pin inside the collagen membrane.

**Figure 1:**
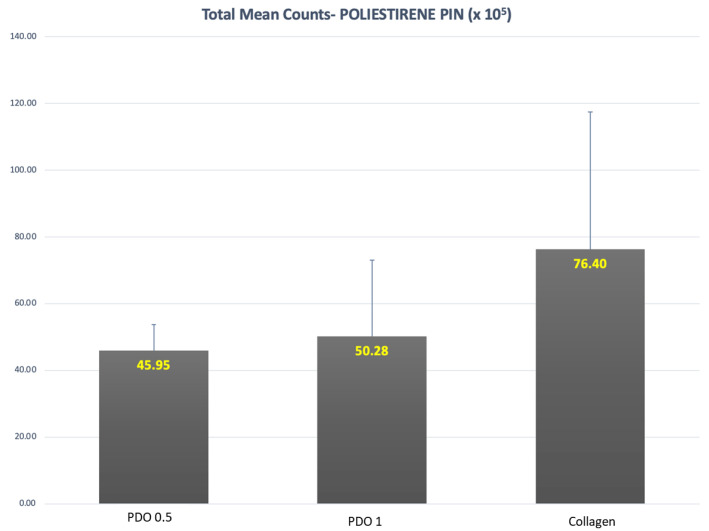
Mean and SD of total count means of the biofilm formed over the polystyrene pins and covered by the evaluated membranes (*p* = 0.08).

In Figure 1, we can see the total count of bacterial species present in the biofilm formed on polystyrene pins inside the membranes. The analysis was performed using checkerboard data, which involved adding up the count of each species within the evaluated groups. Although the pin surrounded by the collagen membrane had about 50% more biofilm compared to the two PDO membranes, there was no statistically significant difference in the average total counts of these three groups (*p* = 0.08).

**Figure 2:**
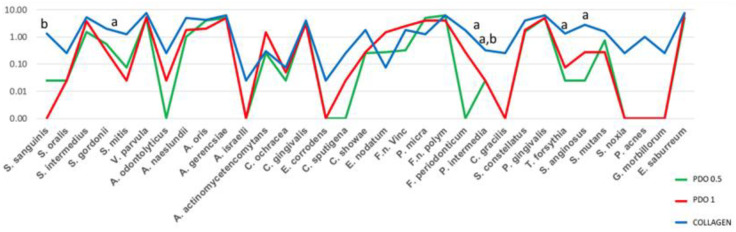
The mean counts of each species formed in the biofilm over the polystyrene pins covered by PDO 0.5, PDO 1.0, and Collagen. The Kruskal–Wallis test and Dunn’s (*p* ≤ 0.05) test were performed. The letter shows the difference between the membranes as follows: “a” shows the difference between PDO 0.5 and collagen; “b” between PDO 1.0 and collagen, and “c” between PDO 0.5 and PDO 1.0.

Figure 2 reveals the results of the biofilm formed over the polystyrene pins covered by the evaluated membranes. Species with significantly different counts (*p* ≤ 0.05) between the internal side of collagen and PDO 0.5 were *S. gordonii*, *F. periodonticum*, *P. intermedia*, *T. forsythia*, and *S. anginosus*, with the polystyrene pins covered by the collagen membrane having a higher count of these bacteria. The species *P. intermedia* had higher significant counts (*p* ≤ 0.05) on the polystyrene pins covered by the collagen membrane compared to PDO 1.

**Figure 3:**
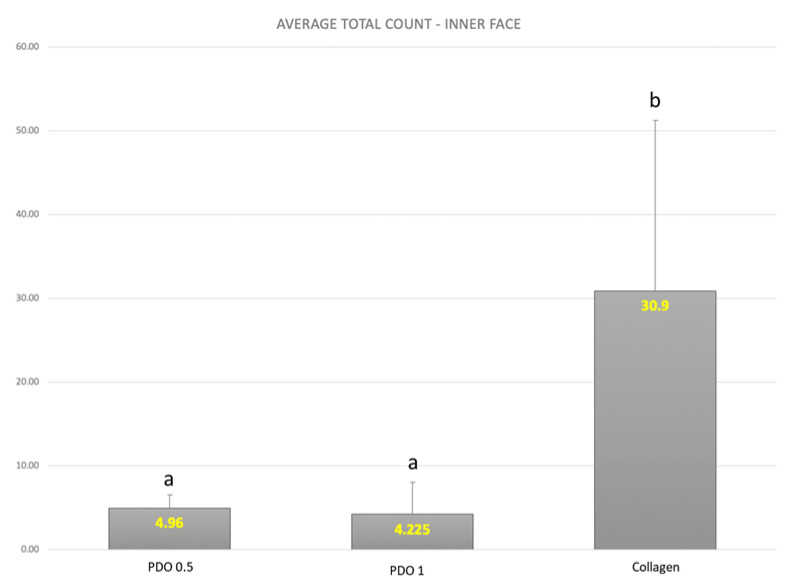
The mean and SD of the total count means of the biofilm formed in the inner part of the polystyrene pins covered by the evaluated membranes. Kruskall–Wallis test (*p* ≤ 0.05) Letters show the difference between the membranes: PDO 0.5 = PDO 1.0 < Collagen.

According to Figure 3, the total biofilm count on the internal side of the collagen membrane was higher than the biofilm counts on both PDO membranes (PDO 0.5 and PDO 1), with a statistically significant difference (*p* ≤ 0.05).

**Figure 4:**
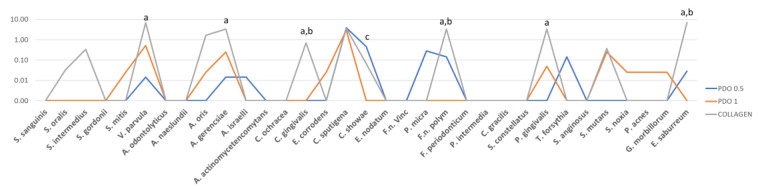
The mean counts of each species formed in the biofilm in the inner face of the PDO 0.5, PDO 1.0, and Collagen. Kruskal–Wallis test and Dunn’s (*p* ≤ 0.05) test were performed. The letter shows the difference between the membranes as follows: “a” shows the difference between PDO 0.5 and collagen; “b” between PDO 1.0 and collagen, and “c” between PDO 0.5 and PDO 1.0.

The results of the biofilm formed on the inner side of the membranes are presented in Figure 4. The average counts of the *V. Parvula*, *A. gerencsiae*, *C. gingivalis*, *F. nucleatum polymorphum*, *E. saburreum*, and *P. gingivalis* species were found to be higher on the inner side of the collagen membrane compared to the same side of the PDO 0.5 membrane (*p* ≤ 0.05). When comparing the two PDO membranes with different thicknesses, it was observed that the thicker one (1 mm) had a statistically lower count of species *C. showae*. Also, when comparing PDO 1 and collagen, a statistically higher count of *C. gingivalis*, *F. nucleatum polymorphum*, and *E. saburreum* was found in the PDO 1 membrane. This suggests that the PDO 1 membrane was more effective in blocking the penetration of significant periodontal pathogens from Socransky’s red complex.

**Figure 5:**
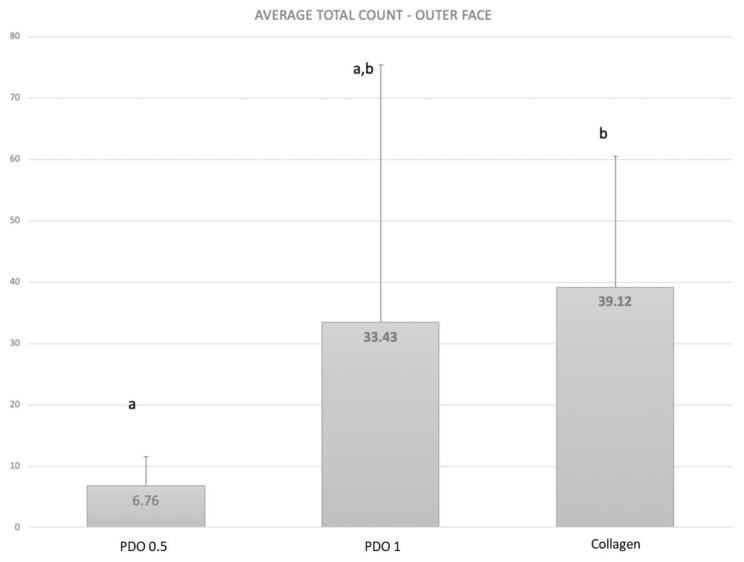
The mean and SD of total count means of the biofilm formed in the external part covered by the evaluated membranes. Kruskall–Wallis test (*p* ≤ 0.05) Letters show the difference between the membranes as follows: PDO 0.5 < PDO 1.0 = Collagen.

According to Figure 5, the PDO 0.5 membrane had less biofilm formation on its external side than the other membrane (*p* ≤ 0.05). However, the biofilm formation on the external side of the PDO 1 mm membrane was similar to the biofilm formed on the other two membranes.

**Figure 6:**
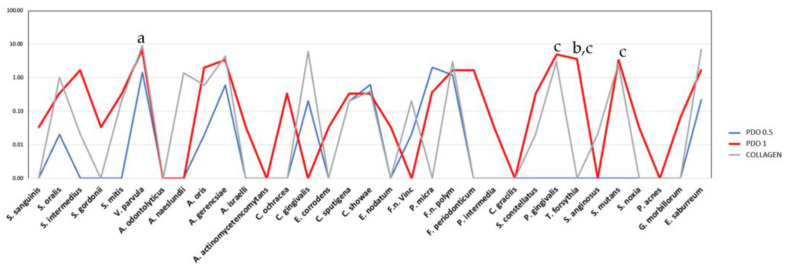
The mean counts of each species formed in the biofilm and formed on the external part of the evaluated membranes PDO 0.5, PDO 1.0, and Collagen. The Kruskal–Wallis test and Dunn’s (*p* ≤ 0.05) test were performed. The letter shows the difference between the membranes as follows: “a” shows the difference between PDO 0.5 and collagen, “b” between PDO 1.0 and collagen, and “c” between PDO 0.5 and PDO 1.0.

In Figure 6, the average count of *V. Parvula* species was found to be higher on the external side of the collagen membrane compared to the external side of the PDO 0.5 membrane (*p* ≤ 0.05). When comparing the two PDO membranes with different thicknesses, it was observed that the thicker one (1 mm) had a statistically higher count of the species *P. gingivalis*, *T. forsythia*, and *S. mutans* (*p* ≤ 0.05). Also, when comparing PDO 1 and collagen, a statistically higher count of *T. forsythia* was found in the PDO 1 membrane (*p* ≤ 0.05).

**Figure 7:**
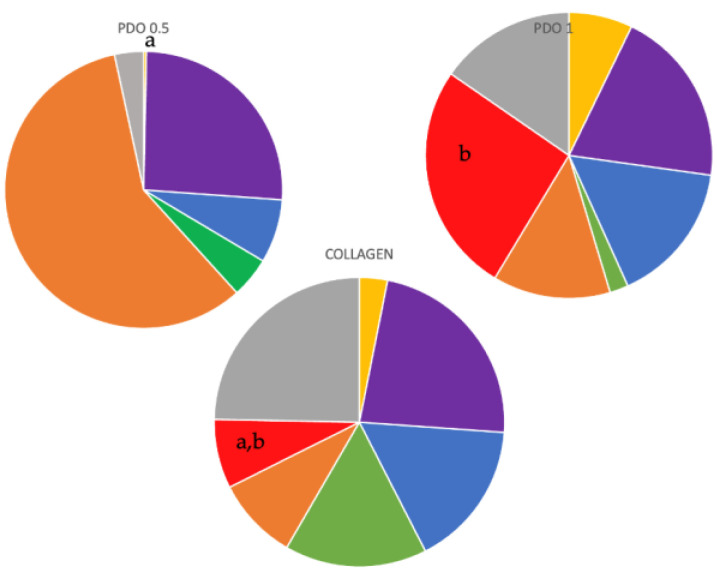
The analysis of microbial complexes of the biofilms formed in the external face of the membranes. The Kruskal–Wallis test and Dunn’s (*p* ≤ 0.05) test were performed. The letters show the differences.

An analysis of complex proportions was conducted for the following three situations: the pin inside the membranes and the internal and external sides of these membranes. For the pin inside and internal side situations, there were no statistical differences observed between any complex in any group. However, for the external sides of the membranes (as shown in Figure 7), the external side of the PDO 1 membrane had a significantly higher proportion than the external side of the PDO 0.5 membrane (*p* ≤ 0.05). No other statistically significant differences were observed between the complexes (*p* ≤ 0.05). Furthermore, the complex proportions of the collagen membrane did not show any significant difference from any other complex of any other membrane.

## 4. Discussion

The data, as illustrated in the charts and the concluding diagram, underscore the pronounced presence of periodontopathogens in the collagen membrane and on the external facet of the PDO 1 membrane. This heightened concentration can be attributed to the colonization prowess of bacteria adept at metabolizing collagen. Specifically, *P. gingivalis* and *T. forsythia* emerged predominantly in the collagen membrane rather than in the PDO 0.5 and PDO 1 membranes.

Contemporary insights into periodontitis underscore the pivotal role of a dysbiotic microbial ensemble in precipitating the disease. Within this cohort, red complex constituents continue to wield a paramount influence over the disease trajectory. Notably, *P. gingivalis* and *T. forsythia* stand out as the most intensely researched bacteria [20,21,22,23,24,25]. These organisms, in light of their potential to shift the oral microbiome from a salubrious state to a diseased one, are earmarked as strategic targets for intervention. Both *P. gingivalis* and *T. forsythia* champion the orchestration of microbial dysbiosis and have intricately evolved strategies to bypass human immunological defenses [24,25,26].

Recent scientific discourse has shone a spotlight on the virulence mechanisms of *Porphyromonas gingivalis* [25]. Owing to its potent pathogenic character, *P. gingivalis* has ascended to the role of a principal protagonist in the narrative of periodontal disease. Gingipains, as a potent subset of virulence factors, have the capability to undermine human immune responses, thereby paving the way for dysbiotic subgingival microbiota to thrive. Additionally, FimA, a fimbrial protein synthesized by *P. gingivalis*, stands out for its ability to stifle the complement system receptor in macrophages [27].

*T. forsythia*, in its own right, has garnered attention as a formidable periodontopathogen [21]. Within its virulence arsenal, the BspA protein has been linked with amplifying alveolar bone degradation during periodontitis episodes. Furthermore, the metabolic by-products stemming from its peptidoglycan breakdown have been flagged for their propensity to destabilize host immune responses, thereby facilitating a dysbiotic subgingival biofilm milieu. Such a landscape of pathogenic microbiota kindles an inflamed reaction, culminating in the sequential degradation of supporting periodontal structures, leading to increased clinical attachment losses and, in grave cases, total tooth loss [2,28].

An illuminating in vitro study identified the pronounced adherence of *P. gingivalis* to collagen membranes [29], vis à vis other membrane types. Compounding this, *P. gingivalis* manifests collagenases that are adept at breaking down collagen membranes. This observation harmonizes with our current study’s insights wherein, post a 7-day incubation window, the collagen membrane exhibited pronounced signs of degradation and wear, juxtaposed with the PDO 0.5 and PDO 1 membranes, which displayed remarkable resilience.

An auxiliary study [30] probing the in vitro permeability and colonization patterns of membranes by *P. gingivalis* postulated that bacterial colonization metrics on regenerative membranes might offer predictive clues about this regenerative treatment’s trajectory. Such insights indicate a compelling need to meticulously manage sites grappling with periodontopathogen infestations to secure regenerative success.

Intriguingly, pathogenic periodontal strains, namely *T. denticola* and *P. gingivalis*, when subjected to in vitro scrutiny, revealed their capacity to bind with various regenerative membranes, culminating in the accelerated degradation of collagen-based membranes [30,31]. Beyond this, these bacteria exhibited a pronounced affinity for collagen membranes. The proteolytic prowess of formidable periodontal adversaries like *P. gingivalis* wields a profound influence on the structural and functional integrity of periodontal cells and tissues [32].

Delving deeper into *P. gingivalis*, its gingipains are identified as the primary catalysts for proteolysis, especially with regard to the collagen-targeting activities of this bacterium. These enzymes, as integral cogs in the bacterial metabolic machinery, possess the capability to degrade a suite of human proteins, encompassing native collagens I, III, IV, and V, fibrin, fibrinogen, fibronectin, protease inhibitors, and immunoglobulins [32,33,34].

The narrative thus far posits PDO membranes as potentially superior candidates if, during clinical use, they result in exposition, given their resistance to microbial degradation. However, the limitation of this in vitro study does not allow us to conclude which membrane should be clinically superior to produce better regenerative results. Nevertheless, while this short-term resistance to bacterial colonization is undoubtedly advantageous, an overarching clinical perspective requires that future endeavors delve deeper and probe longer-term integrative responses and tissue reactions to these membranes to carve out a holistic view of their clinical efficacy. Finally, further clinical comparisons between the membranes must be performed to confirm this in vitro study. 

## 5. Conclusions

The collagen membrane exhibited a higher count of periodontal pathogens. Both the external and internal surfaces of the collagen membrane also demonstrated an increased quantity of periodontal pathogens. Furthermore, the collagen membrane displayed a higher total biofilm count (both internal and external faces) compared to the PDO membranes. Future clinical studies should be performed to corroborate the present data. 

## Data Availability

Data is contained within the article.
